# Monthly migraine days, tablet utilization, and quality of life associated with Rimegepant – post hoc results from an open label safety study (BHV3000–201)

**DOI:** 10.1186/s10194-021-01378-5

**Published:** 2022-01-17

**Authors:** Karissa Johnston, Linda Harris, Lauren Powell, Evan Popoff, Vladimir Coric, Gilbert L’Italien, Curtis P. Schreiber

**Affiliations:** 1 Broadstreet Health Economics & Outcomes Research, Vancouver, BC Canada; 2 Biohaven Pharmaceuticals, New Haven, CT USA; 3 CMH Neurology and Headache Center, Bolivar, Missouri USA

**Keywords:** CGRP, Migraine, Drug treatment, Quality of life

## Abstract

**Background:**

The objective of this study was to describe patterns in monthly migraine days (MMD) and tablet utilization, and to estimate health-related quality of life (HRQoL) measures in patients treated as needed (PRN) with rimegepant 75 mg over 52-weeks.

**Methods:**

Eligible subjects were adults with ≥1 year history of migraine and ≥ 6 MMD at baseline, who used rimegepant 75 mg up to once daily PRN (at their discretion) for up to 52-weeks in an open-label safety study (BHV3000–201; NCT03266588). Mean MMD were calculated at each 4-week period, along with mean monthly tablets taken. Migraine-specific quality of life (MSQv2) data were mapped to EQ-5D utilities and used to characterize HRQoL over time. A published network meta-analysis was used to characterize pain hours as well as time periods spent migraine free.

**Results:**

One thousand forty four subjects were included in this post-hoc analysis. Overall mean MMD were 10.9 at baseline and decreased to 8.9 by week 52. Tablet use remained stable over the follow-up period. A total of 0.08 incremental QALYs were associated with rimegepant use.

**Conclusion:**

For subjects with 6 or more MMD, acute treatment of migraine attacks with rimegepant 75 mg on a PRN basis over one-year of follow-up was found to be associated with reduced MMD frequency without an increase in monthly tablet utilization, and improved HRQoL. There was no evidence of medication-related increases in MMDs when rimegepant 75 mg was used as needed for the acute treatment of migraine over 52-weeks.

**Trial registration:**

ClinicalTrials.gov identifier NCT03266588.

**Supplementary Information:**

The online version contains supplementary material available at 10.1186/s10194-021-01378-5.

## Introduction

Migraine is a common, chronic neurological disorder clinically manifested by severe episodic headaches, resulting in significant burden to patient, health care payers, and society [1, 2]. Migraine is the second-highest cause of disability globally, in individuals across all age groups and becomes more burdensome with increased frequency of monthly migraine days (MMD) [3]. Those with more frequent MMD have reduced quality of life, are less productive, have lower income and higher rates of unemployment, increased disability, increased comorbidity rates, and higher health care resource utilization and direct costs [4–16].

Goals of acute treatment of migraine are to reduce pain, disability, recurrence, and resource use [1]. When first-line agents such as analgesics and triptans are ineffective, intolerable or contraindicated, newer agents such as the calcitonin gene-related peptide (CGRP) receptor antagonist rimegepant are indicated [1]. Rimegepant (BHV-3000) has demonstrated efficacy and safety for the acute and preventive treatment of migraine [17–21], and is approved by the FDA for both indications at the same dose strength (75 mg), with differing administration schedules: as needed (PRN) for acute, and every-other-day (EOD) dosing for prevention [22]. In a long-term (1-year) open-label safety study (BHV3000–201; NCT03266588), a reduction in monthly migraine days (MMD) was observed for patients taking rimegepant on an as needed basis to treat their acute attacks [23]. Other acute migraine therapies, including triptans, have not been associated with a reduction in MMD with long term use [24]. In fact, frequent triptan use may contribute to the development of medication overuse headache, a complex and disabling secondary headache disorder [25]. The long-term health-related quality of life (HRQoL) impact of using a novel acute therapy that also has preventive effects on migraine is currently unknown.

Continued use and reimbursement of novel agents, including rimegepant, is dependent on the rapid and consistent freedom from pain and associated symptoms, restoration of function, and improvements as assessed by a validated patient-reported outcome measure that reflects what patients prefer and value [1]. Measuring HRQoL can be helpful in assessing a treatment’s value for money by providing patient-centered insight about the impact of a treatment (and the disease) on patient functioning, well-being and activities of daily living [26]. HRQoL is very important to patients, as is evident from a study that found that people would prefer to live for 10 rather than 20 years if they were living with a continuous migraine 4.5 days per week [27]. Therefore, reducing the number of days that patients have migraine is paramount for improving HRQoL.

Economic evaluations that assess the value for money of treatments take HRQoL into account in the form of quality-adjusted life years (QALY). QALYs weigh HRQoL against the quantity of years lived in a specific health state [27]. To optimize healthcare resource management, a three-dimensional approach that considers clinical, humanistic and economic outcomes is necessary [28]. Therefore, economic evaluations of migraine treatments require a model that tracks MMD frequency and captures the impact of change in MMD frequency as a result of treatment on resource utilization and QALYs [29].

The least expensive migraine treatment may not always provide the highest value. Less effective treatments may result in other direct and indirect costs, including, increased office and emergency department visits, hospitalization, as well as a humanistic burden affecting HRQoL and causing lost productivity and lost income [28]. For example, a meta-analysis of 56 studies compared eletriptan to sumatriptan, and although sumatriptan was less expensive, eletriptan was more likely to have no adverse events and keep patients pain-free for a sustained period - this resulted in eletriptan having an incremental cost-effectiveness ratio (ICER) of €19,659 per QALY gained compared to sumatriptan [30]. There are a few other economic evaluations available for triptans [31, 32], but triptans have not been demonstrated to decrease migraine frequency [33] and economic evaluations of newer acute treatments for migraine are lacking.

Additionally, clinical trials of the newer treatments most commonly are single-attack study designs which make it difficult to assess the ability of treatments to reduce migraine frequency over time [33]. Therefore, it is currently uncertain whether the ongoing use of new migraine treatments will improve HRQoL over time [33]. The design of the rimegepant long-term safety study, BHV3000–201, offers the opportunity to evaluate this issue. The objective of this study was to describe patterns in MMD, tablet utilization, and estimate HRQoL measures in patients treated over 52-weeks with rimegepant 75 mg oral tablet.

## Methods

### Study design

This was a post-hoc analysis a multicenter, long-term, open-label phase 2/3 safety study of rimegepant 75 mg oral tablet in the acute treatment of migraine (BHV3000–201, NCT03266588) [34, 35]. The study was conducted at 98 sites across the US between August 30, 2017 and July 15, 2019 [34]. A detailed description of BHV3000–201 is provided in Appendix I. Key inclusion criteria were adult patients with at least 1-year history of migraine of moderate or severe pain intensity. Key exclusion criteria included a history of hemiplegic or basilar migraine. Use of prophylactic migraine medications was permitted if the dose had been stable for two months prior to the baseline visit, and the dose was not expected to change during the course of the study. Use of triptans was not permitted during the long-term treatment period.

### Population

Eligible subjects for this post-hoc analysis were a subset of the BHV3000–201 trial, which included adults with a minimum migraine history of one-year year as classified by the International Classification of Headache Disorders, 3rd edition, beta version (ICHD-3 beta), and with ≥6 MMD at baseline [36]. The ≥6 MMD subgroup was selected as these patients were hypothesized to experience a preventative benefit of rimegepant with PRN dosing (in terms of reduced MMD), given the interictal carryover of CGRP antagonism between attacks.

### Intervention

Subjects of this post-hoc analysis treated migraine attacks of any pain intensity with rimegepant 75 mg oral tablet up to once daily as-needed (PRN), for up to 52-weeks [34, 35].

### Outcome measures

The outcome measures of interest for this post-hoc analysis were MMD frequency, the mean monthly number of rimegepant tablets taken, the cumulative pain hours, and impact of migraine on HRQoL. Mean MMD and mean tablet utilization were calculated at each 4-week period. HRQoL over time was characterized by the Migraine-specific quality of life (MSQv2) instrument, which was collected at baseline and weeks 12, 24, 36, and 52.

#### Cumulative pain hours

Cumulative hours of moderate and severe pain, and health-related quality of life (HRQoL) were estimated for patients receiving rimegepant based on BHV3000–201 MMD outcomes and reported HRQoL outcomes and compared to hypothetical outcomes based on baseline clinical status persisting over 52-weeks.

For hours of moderate and severe pain over time, assumed pain trajectories were applied to the MMD values observed over the 52-week follow-up. Pain freedom and relief per migraine event were derived from a network meta-analyses (NMA) conducted by the Institute for Clinical and Evaluative Review (ICER), which compared the proportion of patients experiencing headache relief and pain freedom at 2, 8, 24 and 48 h after treatment with rimegepant or usual care (amongst others) [33]. Pain relief was defined as a decrease in headache pain from moderate tor severe at baseline to mild or no pain at a given follow-up time point (e.g., 2-h) and pain freedom was achieved when a patient was completely pain-free at a given follow-up time point (e.g., 2-h), before the use of rescue medication [33].

Clinical trial data were used to calculate the proportion of patients by pain severity (no pain, mild, moderate, severe) at baseline, 2, 8, 24 and 48 h [33]. Baseline proportions of patients with moderate or severe pain in clinical trials were used as the proportion of patients with migraine, while patients who experienced no pain over a 48-h cycle were classified as patients without migraine [33]. Responses to treatment included no improvement in pain severity at 2, 8, 24 and 48 h, pain relief (improved but not completely resolved), or pain freedom (completely resolved) [33]. Response at 2 h were taken from clinical trials, while responses at 8, 24, and 48 h were estimated by applying non-disclosed risk ratios to responders, or in case of non-responders at 2 h, by applying these risk ratio.to placebo response rates [33, 37].

#### Utility mapping

HRQoL over time was characterized by the MSQv2 instrument, which was collected at baseline and weeks 12, 24, 36, and 52 in BV3000–201. The MSQv2 is a validated 14-question disease-specific patient-reported outcome (PRO) measure that is frequently used to measure the impact of migraine and multidimensional aspects of preventive treatments’ effectiveness on HRQoL in a meaningful way [1, 38]. The MSQv2 has three HRQoL dimensions: Role Function-Restrictive (RR), Role Function-Preventive (RP), and Emotional Function (EF) [39]. The MSQv2 values were mapped to utilities based on a validated algorithm for episodic and chronic migraine [40], and the area under the curve (AUC) of utility values over time was used to calculate quality-adjusted life years (QALYs).

#### Trial oversight/ethics

BHV3000–201 was conducted in accordance with Good Clinical Practice (GCP), and Good Laboratory Practice (GLP), and in compliance with the protocol approved by the Institutional Review Board/Independent Ethics Committee (IRB/IEC), the Declaration of Helsinki, and the Federal Food, Drug and Cosmetic Act and Code of Federal Regulations [41]. Written informed consent was obtained from patients in accordance with IRB/IEC requirements [41].

## Results

BHV3000–201 enrolled 1800 subjects, 1033 (57.4%) in the PRN 2–8 group, 481 (26.7%) in the PRN 9–14 group, and 286 (15.1%) in the QOD + PRN 4–14 group. Overall, the mean (SD) age at migraine onset was 20.6 (10.2) years and the median duration of untreated migraine attacks was 24.0 h. The percentage of subjects with a historic use of discontinued triptans (792 [44.0%]) was comparable with the current use of triptans (741 [41.2%]). For the purposes of BHV3000–201, triptans were discontinued prior to initiating rimegepant. Subjects had history of medical conditions that would be expected in the target population including anxiety (812 [45.1%]), depression (371 [20.6%]) and obesity (321 [17.8%]). A total of 243 (13.5%) of patients used a preventive therapy for migraine if the dose was stable prior to the study start date. The most common therapies were topiramate (118 [6.6%]) and amitriptyline 64 [3.6%]). A small subset of patients were prescribed preventive agents during the course of BHV3000–201 [42, 43].

The analysis described here was conducted in the 1044 subjects with 6 or more MMD from the combined PRN 2–8 and PRN 9–14 study groups. The mean age of the 1044 subjects was 43.2 years and 91.1% of the subjects were female. The mean number of MMD at baseline was 10.9 (Table 1). A total of 635 (61%) completed the 52-week study, while 409 (39%) discontinued.

**Table 1:** Baseline demographics and clinical characteristics

Study	BHV-3000-201
Treatment	Rimegepant
Mean age (years)	43.2
Female (%)	91.1%
Mean MMD at baseline (mean)	10.9

*MMD* monthly migraine day

**Fig. 1:**
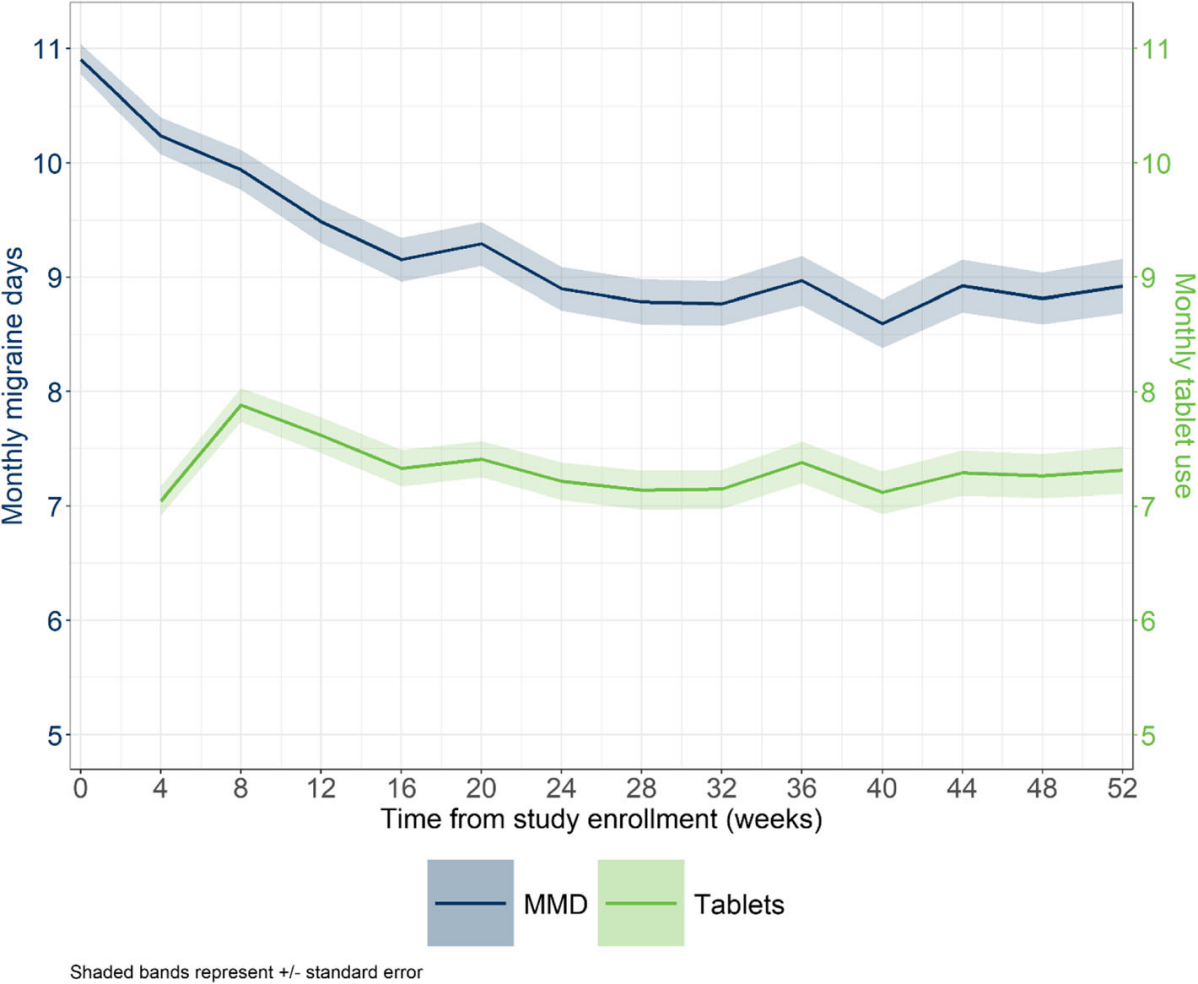
Mean (SE) MMD and PRN rimegepant 75 mg tablet use over time for patients in BHV3000–201. *MMD = monthly migraine days; PRN = as needed*

**Fig. 2:**
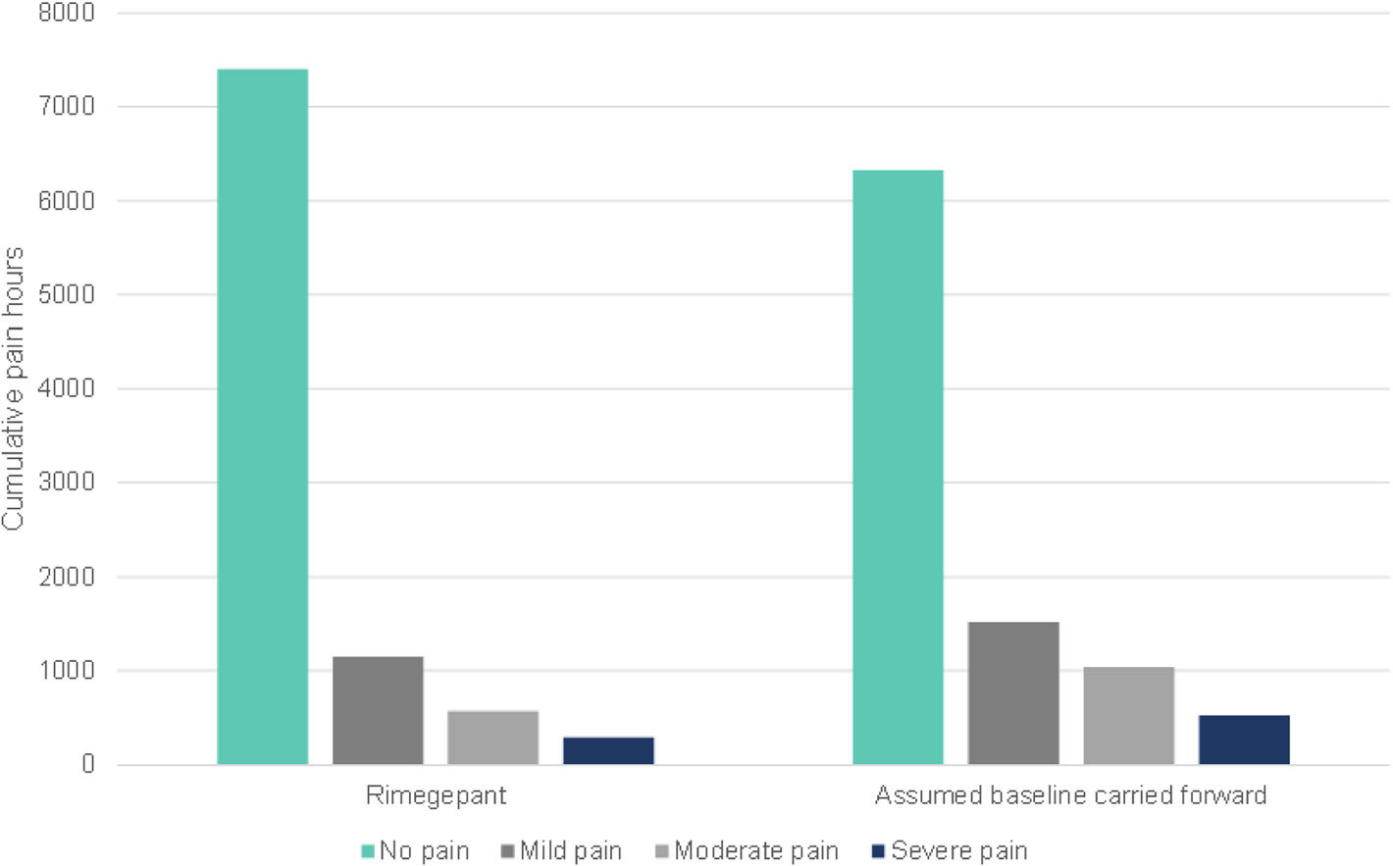
Cumulative pain and pain-free hours calculated over 52-weeks, for two scenarios. Figure 2*illustrates the following two scenarios: 1) patients switch to rimegepant, and 2) patients continue with baseline standard of care treatment*

Among 1044 subjects with ≥6 MMD treated PRN with rimegepant 75 mg tablets, mean MMD were 10.9 at baseline and decreased to 8.9 by week 52. Mean monthly tablet use remained stable with a trend towards decreasing over the period (Fig. 1), from 7.9 tablets in weeks 4–8, to 7.3 tablets in weeks 48–52. Based on this trajectory and estimated pain hours per migraine event (Table 2), rimegepant was estimated to be associated with 16.9% more pain-free hours, 24.4% fewer mild-pain hours, 44.9% fewer moderate pain hours, and 44.7% fewer severe pain hours, compared to continuing the usual care trajectory from baseline to week 52 (Fig. 2).

**Table 2:** Treatment Response in Base-Case ICER Model

Level of Migraine Pain at Time points, %	Rimegepant	Usual Care
Baseline (0 h), %		
None	0.0	0.0
Mild	0.0	0.0
Moderate	66.6	66.6
Severe	33.4	33.4
2 h, %		
None	21.0	11.0
Mild	33.0	24.0
Moderate	30.6	43.3
Severe	15.4	21.7
8 h, %		
None	71.8	53.5
Mild	23.6	32.8
Moderate	3.1	9.1
Severe	1.6	4.6
24 h, %		
None	76.4	68.3
Mild	19.5	21.5
Moderate	2.7	6.8
Severe	1.4	3.4
48 h		
None	82.4	77.4
Mild	12.9	13.6
Moderate	3.1	5.9
Severe	1.6	3.0
**Resulting hours of pain per 48-h migraine event**	**Rimegepant**	**Usual Care**
None	32.5	27.8
Mild	8.9	10.0
Moderate	4.4	6.8
Severe	2.2	3.4

*Source: Atlas* et al. *2020*^*33*^

**Fig. 3:**
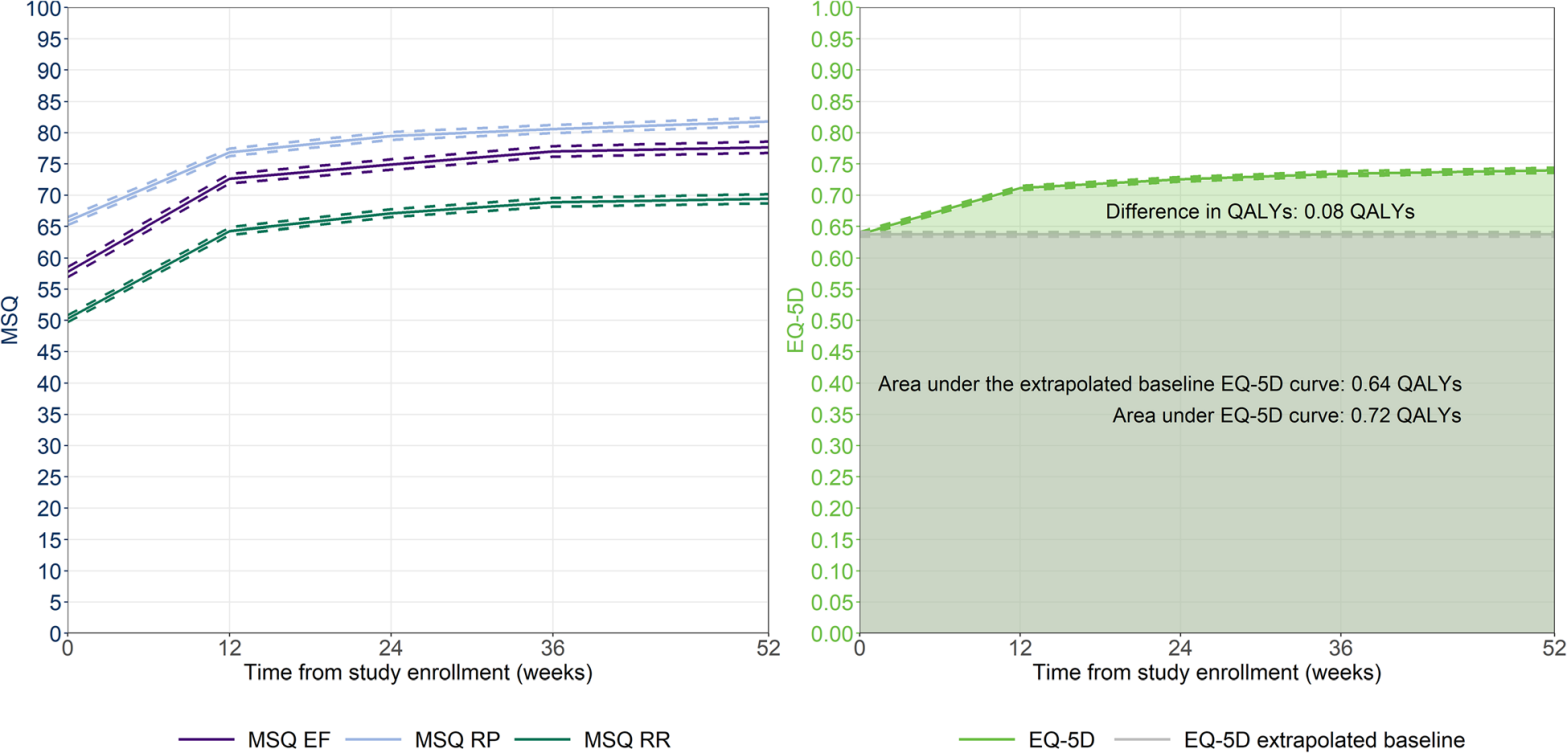
MSQv2 outcomes and mapped utilities for patients in BHV3000–201. *EF = Emotional Function, EQ-5D = EuroQoL five-dimension, MSQ = migraine specific questionnaire, QALY = quality adjusted life year, RP = Role Function-Preventive, RR = Role Function-Restrictive*

MSQv2 improved in all three domains, with a consistent trend of rapid increase in the first 12 weeks, sustained and exhibiting further improvements between weeks 12 and 52 (Fig. 3). When mapped to EQ-5D utilities, the increase was from 0.64 at baseline to 0.75 at week 52, for an estimated cumulative QALY of 0.72. If baseline values were maintained, the QALY over one-year would be 0.64, reflecting an estimated benefit of 0.08 associated with rimegepant use (Fig. 3).

## Discussion

Overall, rimegepant use was associated with a 2.0 MMD reduction from baseline over the 52-week period, and an estimated benefit of 0.08 QALYs. Rimegepant tablet use was stable over time, with no indication of medication-related increases in migraine frequency.

The observed MMD reduction supports the hypothesized that patients with ≥6 MMD at baseline may experience a preventative benefit of rimegepant with PRN dosing, given the interictal carryover of CGRP antagonism between attacks. Alternatively, this observed MMD reduction may be due to more effective acute pain relief with rimegepant (e.g., achieving immediate and sustained pain freedom). For a migraine attack that would last > 24 h if left untreated, successfully aborting the migraine on the first day with rimegepant may contribute to reduced MMD frequency over time.

Meta-analyses, including the ICER review used in this post-hoc analysis, have found that gepants (rimegepant and ubrogepant) are not more effective than triptans for the acute treatment of migraine, and that triptans dominate the newer agents in terms of QALYs (lower total cost with higher QALYs) [33, 44–46]. However, use of triptans have not demonstrated reduced migraine frequency [33], and if change in MMD and delayed benefits are taken into consideration, it may result in a positive assessment of long-term value for money [45]. Additionally, the ICER review did not consider tablet burden, which is particularly relevant given that rimegepant studies only allowed a single dose, while repeat dosing of triptans and other novel acute agents may be needed to achieve a sustained benefit [47–49]. A recent analysis confirmed that when the re-dosing of ubrogepant and lasmiditan was taken into account, rimegepant was associated with lower cost per QALY gained than these other novel agents [50].

Generally, there are few studies that have evaluated the impact of newer acute migraine treatments on MMD over time. In the an open-label study of lasmiditan (the GLADIATOR study) patients treating their migraines with the highest allowed dose of lasmiditan (200 mg) showed a reduction in number of total headache days in the past 3 months (not MMD) from an average of 15.5 days at baseline to 8.2 days at 12 months [33, 51]. Disability was reduced by approximately 50% over a 12 month period, however ≥50% of patients discontinued before the end of the open-label extension study, therefore results should be interpreted in this context [33, 51]. Lasmiditan had higher rates of adverse events compared to rimegepant and ubrogepant. Discontinuation rates during the open-label extension studies due to adverse events were 12.8% for lasmiditan compared to 2.7% for rimegepant and 2.7% for ubrogepant [45].

Ideally, clinical studies should include active treatment comparators to enable direct comparisons between treatments over a long-term period, because indirect comparisons create more uncertainty [33]. Economic evaluations of migraine treatments should take long-term impacts on change in MMD, resource utilization (including pill burden), and medication overuse headaches (MOH) amongst other outcomes into consideration.

Our study is limited by the fact that although BHV3000–201 is a long-term study that provides some evidence of the impact of rimegepant on MMD over time, it is an open-label study with no control group [33]. Placebo effect may play a role in open-label studies and results may be affected by regression to the mean, which makes it impossible to estimate the actual benefit [33]. Another limitation to the study was that the analysis did not control for use of other preventive migraine medications which were allowed in BHV3000–201 when the dose was stable prior to study start date, without protocol violation. However a relatively small proportion (13.5%) of patients in the overall study used these therapies.

There are increased uncertainties with indirect comparisons of treatments compared to direct comparison in RCTs [33]. Limitations of the ICER cost-effectiveness analysis were the heterogeneity among subjects with migraine, lack of reporting on pain severity in clinical trials, and a poor estimation of the magnitude of the delayed effect due to attrition bias and trial design [33]. The ICER review also did not take change in MMD or pill burden into account [33, 49].

Tracking MMD and tablet utilization over a one-year period is a strength as opposed to a single-dose study design where outcomes cannot be tracked over time. There are some advantages to modeling MMD frequency as a continuous outcome, including improved retention of information, increased accuracy of outcome estimates, and between groups comparisons [29].

## Conclusions

Acute treatment of migraine with rimegepant 75 mg on a PRN basis over one-year of follow-up was found to be associated with reduced MMD. In addition there was no increase in tablet utilization frequency, when rimegepant was used on a PRN basis, showing lack of indication for rimegepant to cause medication overuse headache. Reduction in MMD and use of rimegepant to successfully treat the acute migraine episodes jointly resulted in improved HRQoL estimates.

## Supplementary Information

**Additional file 1.** Appendix I – Design of Study BHV3000-201.

## Data Availability

The datasets generated and analyzed for the current study are not publicly available.

## Abbreviations

AE: Adverse effects
AUC: Area under the curve
CGRP: Calcitonin gene-related peptide
EF: Emotional function
EOD: Every-other-day
EQ-5D: EuroQoL five dimension
GCP: Good clinical practice
GLP: Good laboratory practice
HRQoL: Health-related quality of life
ICER: Institute for clinical and evaluative review
ICHD-3: International classification of headache disorders, 3rd edition, beta version
IRB/IEC: Institutional review board/independent ethics committee
MMD: Monthly migraine days
MOH: Medication overuse headaches
MSQv2: Migraine-specific quality of life
NMA: Network meta-analyses
PRN: As needed
QALY: Quality-adjusted life years
QOD: Every other day dosing
RCT: Randomized control trial
RP: Role function-preventive
RR: Role function-restrictive
