# Supplementary material for: Monthly migraine days, tablet utilization, and quality of life associated with Rimegepant – post hoc results from an open label safety study (BHV3000–201)
Source: J Headache Pain. 2022 Jan 17;23(1):10. doi: 10.1186/s10194-021-01378-5 (PMC8903552; doi:10.1186/s10194-021-01378-5)
Supplement: Supplementary file 1 — Additional file 1. Appendix I – Design of Study BHV3000-201. [file 10194_2021_1378_MOESM1_ESM.docx]

**Appendix I – Design of Study BHV3000-201**

Ninety-eight study locations were selected across the US. Recruitment of participants began on August 30th, 2017, with final data collected for the primary outcome measure on July 15th, 2019. The study concluded in August 2019. Key inclusion criteria were male and female participants, 18 years of age or older, with at least a 1-year history of migraine (with or without aura) as defined by the ICHD-3, and at least two to 14 migraine attacks per month of moderate or severe intensity. If concomitant preventative treatments were used, participants had to be on a stable dose (i.e. no change in ≥3 months). Key exclusion criteria included a history of hemiplegic or basilar migraine. Participants were initially screened for study eligibility, followed by a 30-day Observation Period (OP).

In BHV3000-201, there were three enrollment groups based on the self-reported history of moderate to severe pain intensity migraine attacks per month that were treated with rimegepant 75mg as follows: 2-8 attacks/mo, taking rimegepant PRN for up to 52-weeks; 9-14 attacks/mo, taking rimegepant PRN for up to 52-weeks; and 4-14 attacks/mo, taking rimegepant EOD with the option for PRN on non-scheduled days (EOD+PRN) for up to 12-weeks. Note that the EOD dosing group was not included in the current analysis.

For the duration of the OP, participants tracked their migraine severity, frequency, and symptoms daily in an electronic diary (eDiary). MMD were determined as “migraine days per 4-weeks” to match the monthly study visit schedule the participants adhered to. Data was captured in eDiaries by having participants answer “yes” or “no” if they had an attack or not on that day. A migraine day was defined by participants when they answered “yes” in that day’s entry. To keep to the 4-week timeframe, migraine days for the month were calculated within a 28-day period. OP data was considered baseline for participants (Month 0) and following that, 28-day (4-week) intervals from the beginning of the long-term treatment (LTT) period were as follows: Month 1: Weeks 1-4, days 1-28; Month 2: Weeks 5-8, days 29 to 56; and Monthly, up until Month 13: Weeks 49-52, days 336 to 364. Participants progressed to the second study phase, the LTT if they met eligibility criteria and remained compliant with eDiary entries during the OP. The LTT phase was 52-weeks for the subpopulation of interest in the current study.

Table 1 Study BHV3000-201 - Summary of inclusion and exclusion criteria

| **Inclusion Criteria** | |
| --- | --- |
| - participants with 2-14 moderate-to-severe migraine attacks/month - age of onset of migraine prior to 50 years of age - migraine attacks, on average, lasting 4-72 hours if untreated - ability to distinguish migraine attacks from tension/cluster headaches - participants with contraindications for use of triptans may be included provided they meet all other study entry criteria - if using preventive medication, stable dose for ≥3 months | |
| **Exclusion Criteria** |  |
| - history of basilar migraine or hemiplegic migraine - history of HIV disease - history with current evidence of uncontrolled, unstable or recently diagnosed (<6 months prior to enrollment) cardiovascular disease, such as ischemic heart disease, coronary artery vasospasm, and cerebral ischemia. - uncontrolled hypertension or uncontrolled diabetes (however, participants can be included who have stable hypertension and /or diabetes for 3 months prior to screening - history of gastric or small intestinal surgery or has a disease that causes malabsorption - BMI ≥ 30 - HbA1c ≥ 6.5% | |

Abbreviations: BMI = body mass index; HbA1c = hemoglobin A1c; HIV = human immunodeficiency virus

Source: Lipton et al, 2019^29^

***Sample size***

The study sample size determination was based on the ability to rule out adverse events (AEs) that occur at rates greater than 15 cases per 10,000 participants. With a sample size of roughly 2,000 participants, and with no events observed, the upper bound of a one-sided, 95% CI for zero observations is roughly 0.0015. This study had a subpopulation of approximately 800 participants with more frequent migraine attacks that was expected to have a greater exposure to the study drug. With this sample size, and no events observed, the upper bound of a one-sided, 95% CI for zero observations is roughly 0.00375. Hence, this sample size was large enough to rule out AEs that occur at rates greater than 37.5 cases per 10,000 participants.

***Disposition of patients***

Overall, 3,019 participants were screened for study BHV3000-201, 2,867 participants entered the OP, and 1,908 participants subsequently enrolled in the LTT period. The most common reason for not enrolling in the LTT period was screen failure after entering the OP. Among the 1,908 participants enrolled in the LTT period, 1,800 were treated. Among the 108 participants who were not treated after enrolling in the LTT period, the most common reasons for discontinuation were screen failure and withdrawal by participant.

Of the 1,800 treated in the long-term treatment period (LTT) period, 1,197 (66.5%) participants completed treatment (PRN [2-8]: 683 participants; PRN [9-14]: 271 participants; Scheduled EOD + PRN: 243 participants). Among the 603 (33.5%) treated participants who discontinued treatment during the LTT period, the most common reasons overall for discontinuation were noncompliance (184 participants) and withdrawal by participant (159 participants). Other reasons for discontinuation included: lack of efficacy (77 participants); lost to follow-up (76 participants); AE (56 participants); protocol deviation (22 participants); pregnancy (15 participants); screen failure (8 participants); other (5 participants); and positive Sheehan Suicidality Tracking Scale (S-STS) score > 0 (1 participant). Overall mean (SD) time in the LTT period was 42.1 (16.47) weeks for the PRN [2-8] group, 38.0 (18.61) weeks for the PRN [9-14] group, and 11.3 (2.87) weeks for the EOD+PRN group.

***Trial oversight/Ethics:***

Study BHV3000-201 was conducted in accordance with the ethical principles of Good Clinical Practice (GCP), according to the International Conference on Harmonization (ICH) Harmonized Tripartite Guideline, and in accordance with the ethical principles underlying European Union Directive 2001/20/EC, and the United States (US) Code of Federal Regulations (CFR), Title 21, Part 50 (21CFR50). The study was conducted in compliance with the protocol approved by the Institutional Review Board/Independent Ethics Committee (IRB/IEC) prior to initiation of the study. All participants were given a written, informed consent form (ICF) which included all elements required by ICH, GCP, and applicable regulatory requirements. The sample ICF adhered to the ethical principles that have their origin in the Declaration of Helsinki. Biohaven funded this trial and provided the trial drug and conducted the data analyses. A medical writer provided writing and editing services. All authors were involved and responsible for data interpretation, accuracy and completeness, and manuscript preparation and approval.
